# Malaria Liver Stage Susceptibility Locus Identified on Mouse Chromosome 17 by Congenic Mapping

**DOI:** 10.1371/journal.pone.0001874

**Published:** 2008-03-26

**Authors:** Lígia Antunes Gonçalves, Paulo Almeida, Maria Manuel Mota, Carlos Penha-Gonçalves

**Affiliations:** 1 Instituto Gulbenkian de Ciência, Oeiras, Portugal; 2 Unidade de Malária, Instituto de Medicina Molecular, Universidade de Lisboa, Lisboa, Portugal; Leiden University Medical Center, Netherlands

## Abstract

Host genetic variants are known to confer resistance to *Plasmodium* blood stage infection and to control malaria severity both in humans and mice. This work describes the genetic mapping of a locus for resistance to liver stage parasite in the mouse. First, we show that decreased susceptibility to the liver stage of *Plasmodium berghei* in the BALB/c mouse strain is attributable to intra-hepatic factors and impacts on the initial phase of blood stage infection. We used QTL mapping techniques to identify a locus controlling this susceptibility phenotype (LOD score 4.2) on mouse chromosome 17 (*belr1* locus). Furthermore, analysis of congenic mouse strains delimited the *belr1* locus boundaries distally to the H2 region. Quantification of parasites in the liver of infected congenic mice strongly suggested that the *belr1* locus represents a genetic factor controlling the expansion of *P. berghei* in the hepatic tissue. The mapping of *belr1* locus raises the hypothesis that host gene variation is able to control the progression of *Plasmodium* liver stage infection and opens the possibility that the human genomic region orthologue to *belr1* may contain genes that confer resistance to the human malaria liver stage.

## Introduction

Malaria is caused by the hematoprotozoan of the genus *Plasmodium* and provides one of the best examples of how positive selective pressure upon host genetic variants may confer resistance to disease. For example, the mutation underlying sickle cell anemia conferred resistance to malaria (cited in [1]) other erythrocyte traits such as G6PD deficiency, alpha-thalassemia and hemoglobin C are thought to have beneficial effects by reducing parasite invasion or growth in the erythrocytes or by facilitating the elimination of infected erythrocytes (reviewed in [2]). More recently, genome-wide analysis using inbred mouse strains has revealed that a considerable number of chromosomal regions that enhance control of infection with different *Plasmodium* species (reviewed in ([3]).

Genetic analysis of infection with *P. chabaudi* infection has identified nine loci that contribute to control of the parasitemia (*char1–9*) [3], [4]. Analysis of recombinant mouse strains allowed the dissection of multipartite loci in *char2* [5], [6], *char3* [7] and *char4* [8] regions and to the identification of positional candidate genes for the *char9* locus [9]. In particular, a mutation in the pyruvate kinase gene has been identified as mediating a protective mechanism to *P.chabaudi* infection involving increased splenic clearance of erythrocytes through hemolysis [10], [11].

Similarly, resistance to cerebral malaria caused by *P. berghei* infection was mapped to four loci (*berr1*, *berr2*, *cmsc* and a locus on chromosome 18) [12]–[14]. In addition, genetic analysis of the course of *P. berghei* infection identified a locus that controls the resistance to lethal infection (*berr3*) and a locus enhancing survival time (*berr4*) [15]. Parasite clearance and survival to *P. berghei* infection was attributed to a combinatorial effect of *berr1* and *berr3* loci, illustrating that resistance to infection may arise from genetic interaction among host resistance factors [15], [16].

Many malaria-related traits have been analyzed but the role of genetic variants in controlling liver infection has not been addressed. During the initial liver stage of infection, individual sporozoites infect hepatocytes and grow to large schizonts that finally differentiate into 10,000–40,000 merozoites that are released in the bloodstream and infect the red blood cells. A major obstacle to genetic studies in man is the fact that the liver stage infection is largely asymptomatic. Genetic studies in the mouse were initially focused on evaluating the role of MHC alleles in conferring protection induced by irradiated sporozoites [17]. More recently, analysis of knockout mouse models has proved the involvement of host genes like CD81 in the malaria liver stage [18]. As early observations suggested that natural genetic variance between mouse strains may underlie differences in susceptibility to infection with *Plasmodium* sporozoites [19] we have conducted a genetic mapping study to identify genetic factors controlling the liver stage of infection. We report that a quantitative trait locus (*belr1)* mapping to mouse chromosome 17 partially controls expansion of *P.berghei* sporozoites during liver infection.

## Results

### Course of liver stage infection

**Figure 1:**
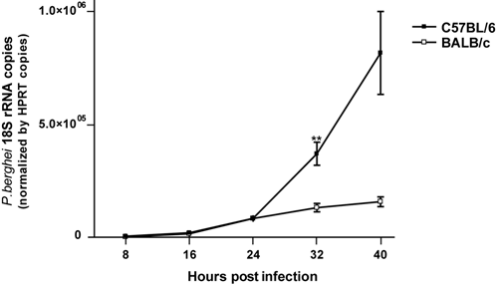
Reduced expansion of *P. berghei* in BALB/c liver after intravenous injection. *P. berghei* 18S rRNA was quantified by real-time PCR in the liver of C57BL/6 and BALB/c males at the indicated times after intravenous injection of 10^4^ *P. berghei* sporozoites. Each data point represents the average value and standard deviation of parasite quantification in the liver of 5–10 mice. Differences between groups were evaluated by unpaired t-test (_**_ p<0.01).

**Figure 2:**
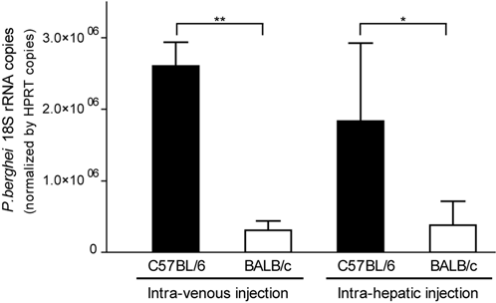
Reduced expansion of *P. berghei* in BALB/c liver after intra-hepatic injection. C57BL/6 and BALB/c mice were surgically injected under the liver capsule with 10^4^ *P. berghei* sporozoites and compared with mice injected intravenously. *P. berghei* 18S rRNA was quantified by real-time PCR at 40 hours after injection. Average values and standard deviations for each experimental group are represented. Differences between groups were evaluated by unpaired t-test (_*_ p<0.05, _**_ p<0.01).

Early reports suggest that infection of BALB/c mice with *P.berghei* sporozoites resulted in low level of hepatocyte infection at the end of the liver stage (44 h post-infection) [19]. We quantified *P.berghei* 18S rRNA in the liver during the course of infection in BALB/c and C57BL/6 mice, after intravenous sporozoite injection. No difference was observed between the two mouse strains during the initial phase of infection but from 24 h up to 40 h post-infection the parasite burden in BALB/c mice was significantly lower (Figure 1). Given that sporozoites invade the liver in less than 1 h, this result suggested that the low parasite expansion in BALB/c mice was controlled within the liver. Next, we quantified the parasite burden in liver of BALB/c and C57BL/6 mice infected by intra-hepatic injection (Figure 2). Once again the parasite burden was lower in BALB/c mice confirming that the poor expansion of *P.berghei* in BALB/c mice was attributable to intra-hepatic factors.

### Liver stage susceptibility phenotype

**Figure 3:**
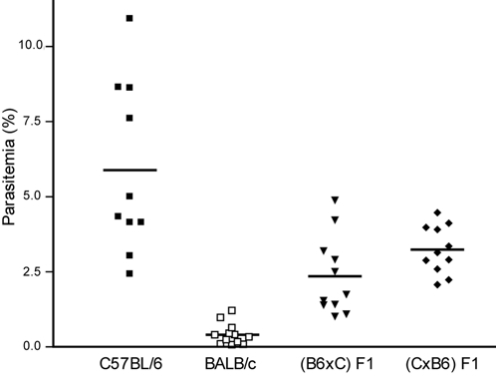
The liver stage susceptibility trait. Parasitemia was measured at day 5 post-infection with 10^4^ *P. berghei* sporozoites in 10 C57BL/6 (dark squares), 12 BALB/c (white squares), 11 (C57BL/6 X BALB/c) F1 (inverted triangles) and 11 (BALB/c X C57BL/6) F1 (diamonds). Group averages are shown as horizontal bars.

**Figure 4:**
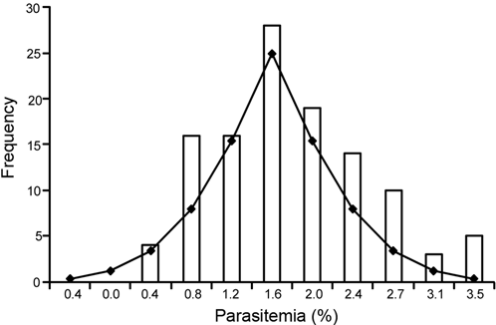
Variance of liver stage susceptibility trait. Frequency distribution of parasitemia at day 5 post-infection in the 115 (C57BL/6 X BALB/c) F2 genotyped in this study. The observed frequencies are represented in bars, and the predicted t-distribution curve is superimposed.

Blood smear analysis of parasitemia rising after sporozoite injection showed that BALB/c mice displayed lower parasitemia than C57BL/6 mice on days 4 and 5 after infection (Figure 3), suggesting that the decreased parasite expansion in the BALB/c delays the development of blood stage infection. Thus, we used parasitemia at day 5 post-infection as an assay to assess the previously occurring parasite expansion in the liver. We studied the segregation of the day-5 parasitemia phenotype in genetic crosses of BALB/c and C57BL/6 mice and we observed that the first generation crosses showed an intermediate phenotype (Figure 3) and that the phenotypic spectrum of the second generation progeny indicated that alleles controlling the phenotype were segregating in the (C57BL/6 X BALB/c) F2 cross. The phenotype in the 115 F2 progeny approached a normal distribution suggesting that could be analyzed as a quantitative trait (Figure 4).

### Genetic mapping of liver susceptibility phenotype

**Figure 5:**
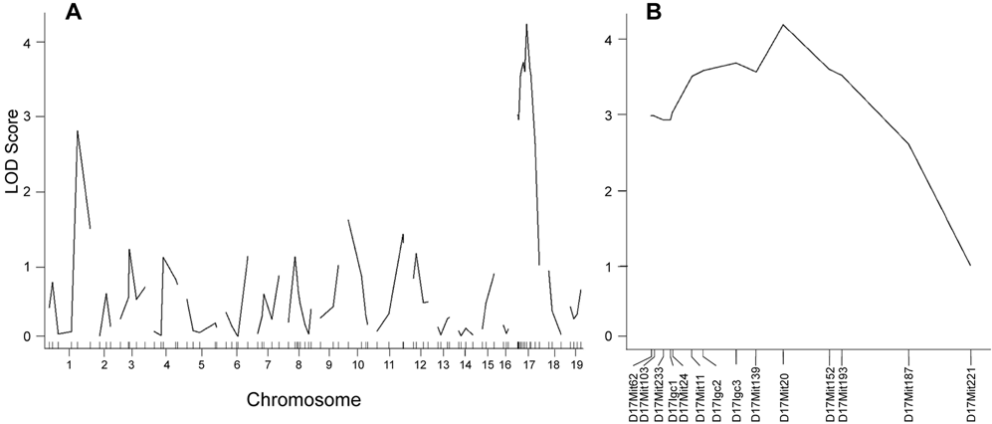
Genome-wide mapping of liver stage susceptibility trait. LOD score curves representing the likelihood for linkage of parasite burden trait. Genome-wide linkage is significant for LOD score above 3.3. (A) Shows LOD score curves for 19 mouse autosomes. (B) Shows LOD score curve for chromosome 17 where the X-axis ticks represent the relative position of microsatellite markers. Markers from left to right: D17Mit62; D17Mit103; D17Mit233; D17Igc1; D17Mit24; D17Mit11; D17Igc2; D17Igc3; D17Mit139; D17Mit20; D17mit152; D17Mit193; D17Mit187; D17Mit221.

In order to map the gene(s) controlling the parasite expansion in the liver, we used 93 microsatellite markers across the whole genome and scanned for quantitative trait loci (QTL) in the 115 (C57BL/6 X BALB/c) F2 progeny. Using QTL statistical analysis, significant linkage was found to the medial region of chromosome 17, where the highest linked marker D17Mit20 reached a LOD score of 4.2 (Figure 5). No other region in the genome reached the genome-wide level of significance, but markers on distal chromosome 1 have shown suggestive linkage (LOD score 2.73), raising the possibility that a second locus mapping in this region may also contribute to the phenotype. The mapped locus in chromosome 17 was named *belr1* locus (*berghei* liver resistance) and the genetic effect of the highest linked marker (D17Mit20) was estimated to explain 15.4% of phenotypic variance observed in the F2 progeny.

### Congenic mapping of *belr1* locus

**Figure 6:**
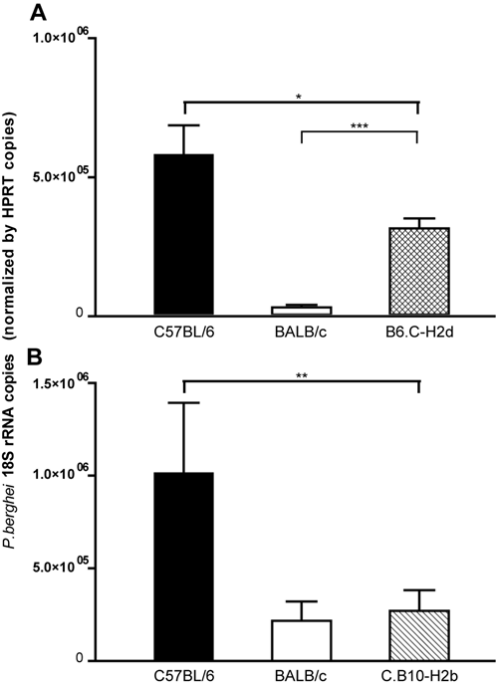
The *belr1* locus controls *Plasmodium* liver burden. *P. berghei* 18S rRNA was quantified by real-time PCR both in the livers of parental mouse strains (C57BL/6 and BALB/c) and in B6.C-H2d congenic mice (A) or in C.B10-H2b congenic mice (B) 40 hours after infection with 10^4^ *P. berghei* sporozoites. The observed difference between C57BL/6 and B6.C-H2d is attributable to the congenic region in chromosome 17 of B6.C-H2d mice while the chromosome 17 congenic region in the C.B10-H2b shows no control over malaria resistance phenotype. Statistical significance was evaluated by unpaired t-test (_*_ p<0.05, _**_ p<0.01, _***_ p<0.001).

**Figure 7:**
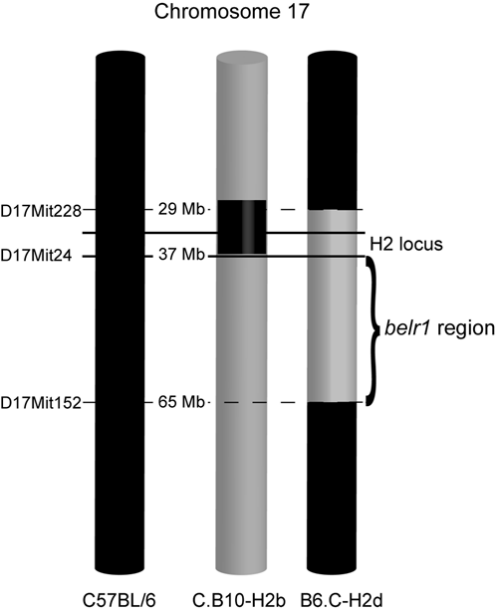
Location of *belr1* locus in chromosome 17. The diagram represents the physical map of chromosome 17 congenic regions in B6.C-H2d and C.B10-H2b mouse strains compared to the C57BL/6 strain. Delimitation of *belr1* locus is represented taking into account the malaria resistance phenotypes in the congenic strains.

To confirm the genetic mapping of the *belr1* locus we analyzed the decreased susceptibility to liver infection in congenic strains containing defined chromosome 17 segments of one parental strain introgressed in the background of the other parental strain. The B6.C-H2d congenic strain carries a 38 Mb segment of BALB/c chromosome 17 encompassing the H2 locus in the genetic background of the C57BL/6 strain. Conversely, the C.B10-H2b strain carries the H2b haplotype in the BALB/c genetic background. We found that the B6.C-H2d strain showed decreased parasite burden in the liver, indicating that the genetic factor mediating the *belr1* effect is contained in this congenic region (Figure 6A). In contrast, C.B10-H2b mice showed liver-stage resistance similar to BALB/c mice (Figure 6B). These results demonstrate that the H2b haplotype *per se* is not controlling the liver burden phenotype, and strongly suggest that the *belr1* locus is mapping distally to the H2 locus. The combined analysis of the congenic strains confines the *belr1* locus to a region of 28 Mb on chromosome 17 distal to the H2 locus (Figure 7). As an alternative approach, we performed a conditional analysis in the F2 progeny that fixed the H2 genetic effect by using the D17MIT233 marker as a co-variate in the QTL analysis. Under this analysis the LOD score curve still persisted on the *belr1* locus albeit with lower values. These results suggest that the *belr1* genetic effect is independent of the H2 locus, but do raise the possibility that genes in the H2d haplotype present in the B6.C-H2d mouse strain may also contribute to the malaria liver-stage resistance.

Collectively, the results indicate that a genetic factor(s) controlling susceptibility to liver infection by *P. berghei* is located in the *belr1* locus and acts at the intra-hepatic level to control parasite liver stage expansion.

## Discussion

The key observation in this work is the demonstration that the *belr1* locus controls the expansion of *P. berghei* in the liver, and that impacts on the level of subsequent blood parasitemia. Using mouse congenic strains the locus controlling parasite expansion in the liver was delimited within an interval of 28 Mb on mouse chromosome 17 close to the MHC locus previously linked to malaria resistance in both man and mouse. Although this analysis indicated that the major genetic effect on chromosome 17 maps outside the H2 region it is still possible that the H2 locus could contribute to the observed phenotype. In addition, genetic markers in distal chromosome 1 suggest that a second locus could also be involved in the genetic control of this phenotype. Incidentally, this region coincides with a locus (*berr1*), that we have previously shown to control cerebral malaria and parasitemia clearance following *P. berghei* ANKA infection [12], [15]. To further dissect such multiple genetic effects will include the generation and analysis of subcongenic strains carrying different congenic intervals derived form the B6.C–H2d mouse strain.

The possible involvement of the MHC region in the genetic control of resistance to malaria resistance in humans has stimulated a search for the contribution of the MHC and TNF molecules to the pathogenesis of malaria. For example, a significant number of reports claim genetic linkage and allelic association of these genes with severe forms of disease and re-infection risk [20]–[24]. At present, it is difficult to determine whether the genetic effects presented in these studies could result from genetic factors mapping just outside the MHC region like the *belr1* locus. It is of immediate interest to evaluate the human orthologue to *belr1* region to malaria genetic resistance in humans through unbiased association mapping studies.

Previous genetic analysis determined that mouse chromosome 17 contains loci exerting weak control of resistance to *P. chabaudi* parasitemia [25] , in particular the H2-linked locus, *char3*, which mediates delayed parasitemia kinetics [26]. Further work suggested that these genetic effects were possibly contributed by an additional locus, *char7*, mapping distally to the H2 in a region that co-localizes with *belr1* locus [7]. Thus, it remains to be determined whether the locus described in this report coincides with *char7* locus, thereby representing a genetic factor controlling malaria infection at both liver and blood stages and having a relevant role in the disease caused by different murine *Plasmodium* species.

The *belr1* region contains 384 genes and includes known genes, predicted genes and other ORFs. Certainly, some genes are readily excluded from the liver resistance phenotype, as is the case of 49 genes coding for olfactory receptors. However, the presence of at least 80 unknown genes precludes the identification of plausible candidates. Subcongenic mapping will be performed to narrow down the number of credible positional candidate genes which will then be subjected to functional and structural analysis. The identification of candidate genes for the *belr1* locus may lead to the characterization of similar human genetic factors playing a role in malaria resistance by though their impact on asymptomatic liver infection.

It is likely that cell and molecular components that are involved in the observed host resistance exert their effect by limiting the expansion of the parasite liver stages in the hepatic tissue, raising the question of the underlying resistance mechanism. One possibility is suggested by work with *P. berghei* [19] where the blockage of liver-stage parasite development in BALB/c liver has been correlated with the immediate liver inflammatory response [27]. However, the relevant effectors and target cells have not been defined. One attractive hypothesis is that, liver resident cell types, such as Kupfer cells or NK and NKT cells act on the parasite, in either extracellular or intracellular forms.

Alternatively, the observed resistance to *Plasmodium* expansion could be mediated by a hepatocyte factor that controls the survival of infected hepatocytes, for example sporozoite mediated inhibition of hepatocyte apoptosis. In fact, release of hepatocyte growth factor (HGF) [28] and signaling through the HGF/c-Met pathway [29] are considered as part of a chain of events that leads to apoptosis resistance of *P. berghei*-infected hepatocytes [30]. It has been shown that the initial protection of the host cell from spontaneous apoptosis mediated by HGF/c-Met signaling is extended in time possibly by induction of additional apoptosis resistance mechanisms [31]. We are currently testing this possibility by studying the induction of hepatocyte apoptosis in the presence of *P. berghei* sporozoites.

Searching for such cellular phenotypes in relevant congenic strains is part of a strategy to identify the cell type(s) mediating the *belr1* effect. If successful, this approach will accelerate the identification of the *belr1* gene(s).

## Materials and Methods

### Mice

Parental mouse strains used in this study were C57BL/6, BALB/c, (BALB/c X C57BL/6) F1 and (C57BL/6 X BALB/c) F1. (C57BL/6 X BALB/c) F1 mice were used to generate the (C57BL/6 X BALB/c) F2 progeny of 115 males. Two H2 congenic mouse strains were obtained from the Jackson Laboratory (Maine, USA) and maintained at IGC. B6.C-*H2^d^*/bByJ carries a BALB/c-derived congenic segment of 37.9 Mb on chromosome 17, flanked by markers D17Mit198 and D17Mit152, on C57BL/6 genetic background and is here referred to as B6.C-H2d. C.B10-*H2^b^*/LilMcdJ carries a C57Bl/10-derived segment of 10.4 Mb on chromosome 17, flanked by markers D17Mit80 and D17Mit232, on BALB/c genetic background and is here referred to as C.B10-H2b. The haplotype b of H2 is common to strains C57BL/6 and C57BL/10. All mice were bred and maintained in conventional housing facilities at the Instituto Gulbenkian de Ciência. All experiments used male mice with 8 to 15 weeks of age. All procedures were in accordance with national regulations on animal experimentation and welfare.

### Infection

*Plasmodium berghei* ANKA sporozoites were obtained from dissection of salivary glands from infected female *Anopheles stephensi* mosquitoes. Sporozoites suspensions in RPMI medium were injected i.v. in 100 µl inocula of 10^4^ sporozoites per mouse.

To perform the intra-hepatic injection, the mice were anesthetized with Ketamine by i.p. injection. The abdominal cavity was surgically accessed through the medial line, partially exposing the left liver lobe. Sporozoites were injected under the liver capsule in a 50 µl inoculum. Then, the abdominal wall muscles and skin were sutured, and the animals were maintained until 40 h post-infection.

### Parasite quantification

Parasitemia progression was measured at day 5 post-infection by blood smears with Giemsa. Liver parasite 18S rRNA was quantified by real-time PCR. Livers were collected at 40 h post-infection, immediately homogenized in denaturing solution (4 M guanidine thiocyanate, 25 mM sodium citrate pH 7.0, 0.5% sarcosyl and 0.7% β-mercaptoethanol in DEPC treated water) and total RNA was obtained using RNeasy Mini Kit (Qiagen). One microgram of total RNA was converted to cDNA (Transcriptor First Strand cDNA Synthesis Kit, Roche) and cDNA specific to *P. berghei* 18S rRNA was amplified with primers NYU-Pb1 5′-AAG CAT TAA ATA AAG CGA ATA CAT CCT TAC-3′ and NYU-Pb2 5′-GGA GAT TGG TTT TGA CGT TTA TGT-3′. The real-time PCR reactions were performed in ABI Prism 7900HT system using ABI Power SYBR Green PCR Master Mix. Absolute *P.berghei* 18S rRNA estimates were normalised for mRNA of Hypoxanthine Guanine Phosphoribosyl-Transferase (HPRT), a mouse housekeeping gene.

### Genotyping

Genomic DNA was prepared from mouse tails before infection, using standard techniques. The 115 (C57BL/6 X BALB/c) F2 progeny mice were genotyped by using conventional PCR protocols for 93 microsatellite markers obtained from the Whitehead/MIT Center for Genome Research collection (www.genome.wi.mit.edu/cgi-bin/mouse/index). Primers for Igc markers were: D17Igc1 For GGG AGT GGG AAT TCT TTT ATT TTA and Rev TGC TTT CTT CTG GTG TCT CTG AA; D17Igc2 For GCT CAC TTT TTC CTA GCA TCA TC and Rev GCC ATG GGA AGA AGT TAT ATG TC; D17Igc3 For GAT AAG TTT GGA GTC AGG CCT AA and Rev ACT TAT TCA CTC CTG AGC CTT GT. Individual mouse genotypes were scored using established agarose gel electrophoresis protocols.

### Statistical Analysis

Quantitative trait locus analysis was performed using the R/QTL software [32]. This program calculates logarithm of odds (LOD) scores over intervals between linked markers, generating LOD score curves representing the likelihood of genetic linkage of quantitative phenotypes with markers along the chromosome. The level of statistical significance was empirically determined by permutation tests (5.000) and genome-wide significant linkage was considered when LOD ≥3.3 (p<0.05).

Comparisons between groups of animals were considered statistical significant when the p-value of the unpaired t-test was <0.05.
